# An Updated Evidence About the Role of Timing to Debridement on Infection Rate of Open Tibial Fractures: A Meta-Analysis

**DOI:** 10.7759/cureus.10379

**Published:** 2020-09-11

**Authors:** Ahmed Elnewishy

**Affiliations:** 1 Orthopaedic Surgery, Kasr Al-Ainy Medical School, Kafr El Sheikh, EGY

**Keywords:** early debridement, open fractures, tibia, meta-analysis.

## Abstract

Background and objectives

Recent experimental and clinical evidence supporting early debridement for open fractures has been questioned. Therefore, this systematic review and meta-analysis aimed to summarize and evaluate the current evidence regarding the timing of surgical debridement of open tibial fractures.

Methods

A systematic review and meta-analysis were conducted on studies compared the infection rate following early versus late debridement of open tibial fractures. We performed an online, bibliographic, search through the period from January 2000 to June 2020 in five bibliographic databases: Cochrane Central Register of Controlled Trials (CENTRAL), Medline via PubMed, Web of Science, Scopus, and EBSCO host.

Results

Nine retrospective studies and six prospective studies were included in the present meta-analysis study. The pooled effect estimate showed no statistically significant difference between early and late debridement regarding the overall infection rate (RD 0.02, 95% CI [0 - 0.04], p = 0.94); there was no significant heterogeneity in the pooled estimate (I2 = 5%). The subgroup analysis showed that the non-significant difference was consistent regardless of the definition of early and late timing to debridement. Likewise, the pooled effect estimate showed no statistically significant difference between early and late debridement regarding the deep infection rate (RD 0.01, 95% CI [-0.01 - 0.03], p = 0.92); there was no significant heterogeneity in the pooled estimate (I2 = 0%). The pooled effect estimate showed no statistically significant difference between early and late debridement regarding the nonunion rate as well. The funnel lots showed little evidence of asymmetry by visual inspection.

Conclusion

In conclusion, the current evidence demonstrates no impact of timing to surgical debridement on the infection rate following open tibial fractures in the adult population. Our results demonstrated that the risks of infection, deep infection, and nonunion were similar between patients who underwent delayed versus early debridement.

## Introduction

Tibia fractures are the most common long bone fractures with an infection rate of 20 times higher than other open long bone fractures [1]. According to the epidemiologic studies, the incidence rate of open long bone fractures is 11.5 per 100,000 persons annually [2]. It was estimated that the prevalence of infection following internal fixation of fractures could reach up to 30% in open fractures. Several tissue damage and wound contamination can lead to bone and soft tissue necrosis and infection due to open fractures. In severe cases, dysfunction of the limb and several organ failures can occur [3].

The number of open fractures and similar high-energy injuries has increased despite the advances in antibiotics, fracture stabilization, and wound management, which dramatically decreased the mortality from open fractures. Therefore, many investigators were proposed urgent operative debridement of open tibial fractures along with early administration of antibiotics [4-6]. In order to reduce the risk of infection and nonunion, it has been recommended that open tibial fractures should be debrided within 6 hours from injury, which is known as the 6-hour rule [7]. The 6-hour rule comes from a study of Friedrich that was conducted on guinea-pigs and showed that all animals remained healthy when debridement of open wounds was performed within 6 hours [8]. However, if the time after injury is >24 h, debridement is not recommended due to bacterial multiplication. In medical practice, several external variables will delay the timing of debridement in emergency surgery, including the delay in delivery, the patient's unstable condition, and other combined injuries being treated urgently [9]. Hence, debriding some patients within 6 hours after the injury is difficult. Despite these recommendations, some retrospective studies suggest that time to debridement is not a major determinant of the outcome [10, 11]. Moreover, the experimental and clinical evidence supporting this recommendation has been questioned [12-15]. Therefore, this systematic review and meta-analysis aimed to summarize and evaluate the current evidence regarding the timing of surgical debridement of open tibial fractures.

## Materials and methods

We followed the recommended standards provided by the second edition of the Cochrane Handbook for Systematic Reviews of Intervention during the conduction of the present systematic review [16]. The writing of the present manuscript was done in strict adherent to the Preferred Reporting Items for Systematic Reviews and Meta-Analyses (PRISMA) statement [17].

Eligibility criteria

Studies in English language were deemed eligible for the present systematic review if they met all of the following criteria: 1) adult patients (≥18 years old) with open tibial fractures; 2) studies which compared the infection rate following early versus late debridement of open tibial fractures; and 3) prospective or retrospective studies were included. We excluded studies with duplicate dataset, narrative or systematic reviews, studies with no data regarding the infection rate, animal models, studies in which open tibial fractures represent less than 50% of the total number of included fractures, dissertations, and conference abstracts.

Literature search strategy and screening

We performed an online, bibliographic, search through the period from January 2000 to June 2020 in five bibliographic databases: Cochrane Central Register of Controlled Trials (CENTRAL), Medline via PubMed, Web of Science, Scopus, and EBSCO host. Various combinations of the following queries were utilized: Tibial fractures, open fractures, debridement, timing to debridement, infection rate. In order to remove duplicates from databases search, we downloaded the retrieved citations and imported them to EndNote X7 for duplicates removal. Then, the titles and abstracts of the remaining records were screened for eligibility. A second-round of screening was conducted on full-texts of potentially eligible abstracts for final inclusion in the present systematic review.

Data extraction

We developed a standardized data extraction form using Excel software for data retrieval and processing. The following data were extracted from each eligible study: first author name, year of publication, study design, number of participants, number of fractures, timing to debridement, main findings, age, gender, Gustilo grading, overall infection rate, deep infection rate, and non-union rate. The quality assessment of the included studies was done using Newcastle-Ottawa Scale (NOS) [18].

Data analysis

All data were analyzed using STATA version 16.0 (StataCorp LLC, College Station, TX). The pooled estimates of risk difference (RD) were calculated using random effect models with inverse variance weighting. The primary data for overall infection, deep infection and non-union (event and non-event) from each included article were used to estimate the risk difference for each study. Heterogeneity among included studies was assessed based on the visual examination and Cochrane Q and the I2 statistics. Subgroup analysis was conducted to assess the risk of timing the primary outcomes. All findings were presented as RD with 95% confidence interval (95% CI). P value less than 0.05 was considered significant.

## Results

**Figure 1:**
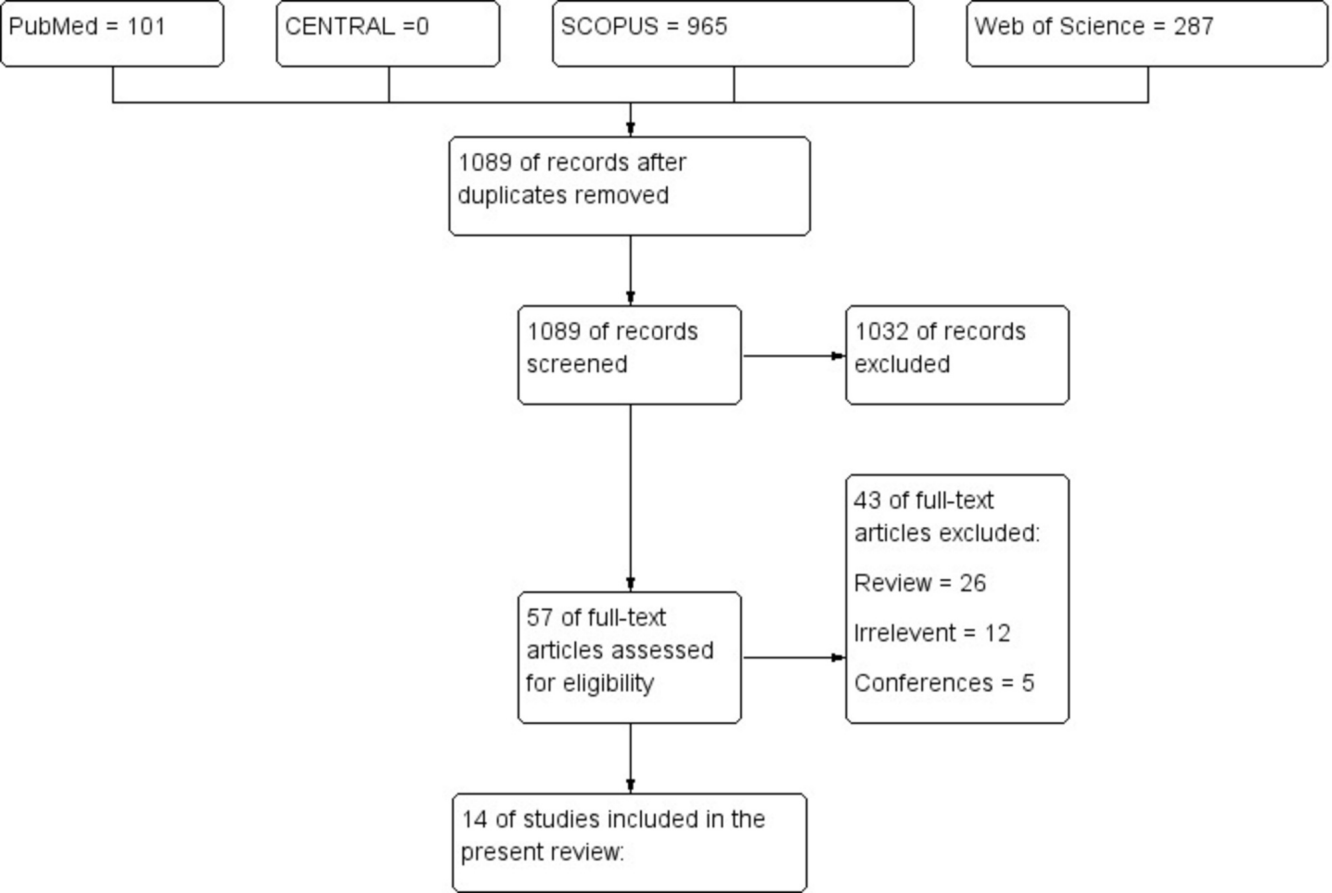
PRISMA flow diagram

A total of 1353 records were retrieved from an online search. Of them, 1089 records were screened after duplicates removal. After the initial screening, 57 full texts were retained for a full evaluation. Out of them, 43 studies were excluded as they were narrative or systematic review (n = 26), irrelevant (n = 12), and conferences (n = 5). Finally, a total of 15 studies (prospective studies = 6; retrospective studies = 9) were included in the present systematic review (See PRISMA flow diagram; Figure 1).

Nine retrospective studies [4, 6, 11, 12, 19-23] and six prospective studies [3, 5, 10, 15, 24, 25] were included in the present meta-analysis study. The number of fractures ranged from 41 to 383 fractures. The majority of fractures within the included studies were middle-third fractures. Nine studies compared early debridement within six hours from fracture to late debridement beyond those six hours, two studies compared early debridement within eight hours to late debridement beyond the eight hours, and one study compared < five hours to ≥ five hours and < 12 hours to ≥12 hours, each. In Li et al. and Srour et al. studies, multiple timings to debridement were compared (Table 1). The clinical characteristics of the included studies are shown in Table 2.

**Table 1:** Summary of the included studies LFITFD: Length from injury to first debridement

Author	Year	Study design	Type of fracture			No. of patients	No. of fractures	Early debridement	Late debridement	Main findings	Level of evidence
			Middle	Proximal third	Distal						
Charalambous et al.	2005	Retrospective	NA	NA	NA	383	383	≤ 6 Hours	> 6 Hours	No relation between debridement timing and infection rate	III
Khatod et al.	2003	Retrospective	47	27	40	103	101	≤ 6 Hours	> 6 Hours	No relation between debridement timing and infection rate	III
Spencer et al.	2004	Retrospective	41	0	0	.	41	≤ 6 Hours	> 6 Hours	No relation between debridement timing and infection rate	II
Sungaran et al.	2007	Retrospective	161	0	0	161	161	≤ 6 Hours	> 6 Hours	No relation between debridement timing and infection rate	III
Kamat	2011	Retrospective	103	0	0		103	≤ 6 Hours	> 6 Hours	No relation between debridement timing and infection rate	III
Enninghorst et al.	2011	Prospective	89	0	0	89	89	≤ 6 Hours	> 6 Hours	Time to debridement is a predictor of poor outcome	II
Singh et al.	2012	Prospective	25	8	34	67	67	≤ 6 Hours	> 6 Hours	No relation between debridement timing and infection rate	II
Reuss and Cole	2007	Retrospective	61	5	15	77	81	< 8 Hours	> 8 Hours	No relation between debridement timing and infection rate	III
Harley et al.	2002	Retrospective	NA	NA	NA	NA	89	≤ 8 Hours	> 8 Hours	No relation between debridement timing and infection rate	III
Fernandes et al.	2015	Prospective	NA	NA	NA	NA	76	< 6 Hours	> 6 Hours	A significant increase in the rate of infection was observed in those operated 6 hours after trauma.	II
Hendrickson et al.	2018	Retrospective	NA	NA	NA	112	116	< 12 Hours	> 12 Hours	No relation between debridement timing and infection rate	III
Li et al.	2020	Retrospective	74	48	93	215	215	≤ 6 Hours	6 h < LFITFD ≦ 12 h or 12 h < LFITFD ≦ 24 h	No relation between debridement timing and infection rate	III
Pollak et al.	2010	Prospective	NA	NA	NA	307	307	< 5 Hours	> 5 Hours	No relation between debridement timing and infection rate	II
Srour et al.	2015	Prospective	NA	NA	NA	64	64	<6 Hours	7 to 12 hours; 13 to 18 Hours; or 19 to 24 Hours	No relation between debridement timing and infection rate	II
Al-Arabi et al.	2007	Prospective	NA	NA	NA	237	248	< 6 Hours	> 6 Hours	No relation between debridement timing and infection rate	II

**Table 2:** Baseline characteristics of the included studies LFITFD: Length from injury to first debridement

Author	Group	Mean age	Males	No. of fractures	Gustilo grading				
					1	2	3A	3B	3C
Charalambous et al.	Early	31 (Range 4-87)	32	184	14	19	109	42	0
	Late	30 (Range 3-88)	30	199	19	19	139	22	0
Khatod et al.	Early	34 (Range 6-90)	NA	73	12	37	12	5	7
	Late		NA	30	7	9	11	3	0
Spencer et al. (35%)	Early	NA	NA	27	5	4	8	9	NA
	Late	NA	NA	14	5	1	6	2	0
Sungaran et al.	Early	NA	NA	65	7	10	48		
	Late	NA	NA	96	21	25	50		
Kamat et al.	Early	NA	NA	62	19	21	12		
	Late	NA	NA	41	30	11	10		
Enninghorst et al.	Early	41 + 7	66	46	NA	NA	NA	NA	NA
	Late			43	NA	NA	NA	NA	NA
Singh et al.	Early	32.4 (Range 7-89)	54	38	0	0	38		
	Late			29	0	0	29		
Reuss and Cole	Early	NA	23	31	5	5	2	15	4
	Late	NA	40	50	9	14	7	19	1
Harley et al.	Early	NA	NA	41	19	53	37		
	Late	NA	NA	48					
Fernandes et al.	Early	NA	NA	NA	NA	NA	NA	NA	NA
	Late	NA	NA	NA	NA	NA	NA	NA	NA
Hendrickson et al.	Early	47 (Range 18-98)	NA	44	0	0	0	44	0
	Late	53 (Range 17-93)	NA	72	0	0	0	72	0
Li et al.	≤ 6 Hours	48.5 + 3.6	117	65	62	98	26	25	4
	6 h < LFITFD ≦ 12 h			95					
	12 h < LFITFD ≦ 24 h			36					
	LFITFD > 24 h			19					
Pollak et al.	Early	(Range 16-69)	NA	93	NA	NA	NA	NA	NA
	Late		NA	214	NA	NA	NA	NA	NA
Srour et al. (48.3%)	<6	37.0 (17.2)	46	64	9	22	18	9	6
	7 to 12 Hours	33.8 (15.8)	54	70	13	24	22	8	3
	13 to 18 Hours	32.4 (17.8)	81	98	33	28	23	10	4
	19 to 24 Hours	33.4 (14.2)	68	83	15	20	32	14	2
Al-Arabi et al. (< 50%)	Early	41	NA	154	77	54	65	52	0
	Late		NA	94					

With regard to the risk of bias, all prospective studies reported adequate selection of the cases; the comparability was adequate in most of the prospective studies as well. The drop-out rate was adequate in all included, prospective, studies; however, no clear descriptions were provided regarding the method of assessment of infection. The overall quality of the prospective studies was moderate-to-high. On the other hand, the selection, comparability, and exposure domains were deemed adequate in most of the included retrospective studies (Appendix 1).

**Figure 2:**
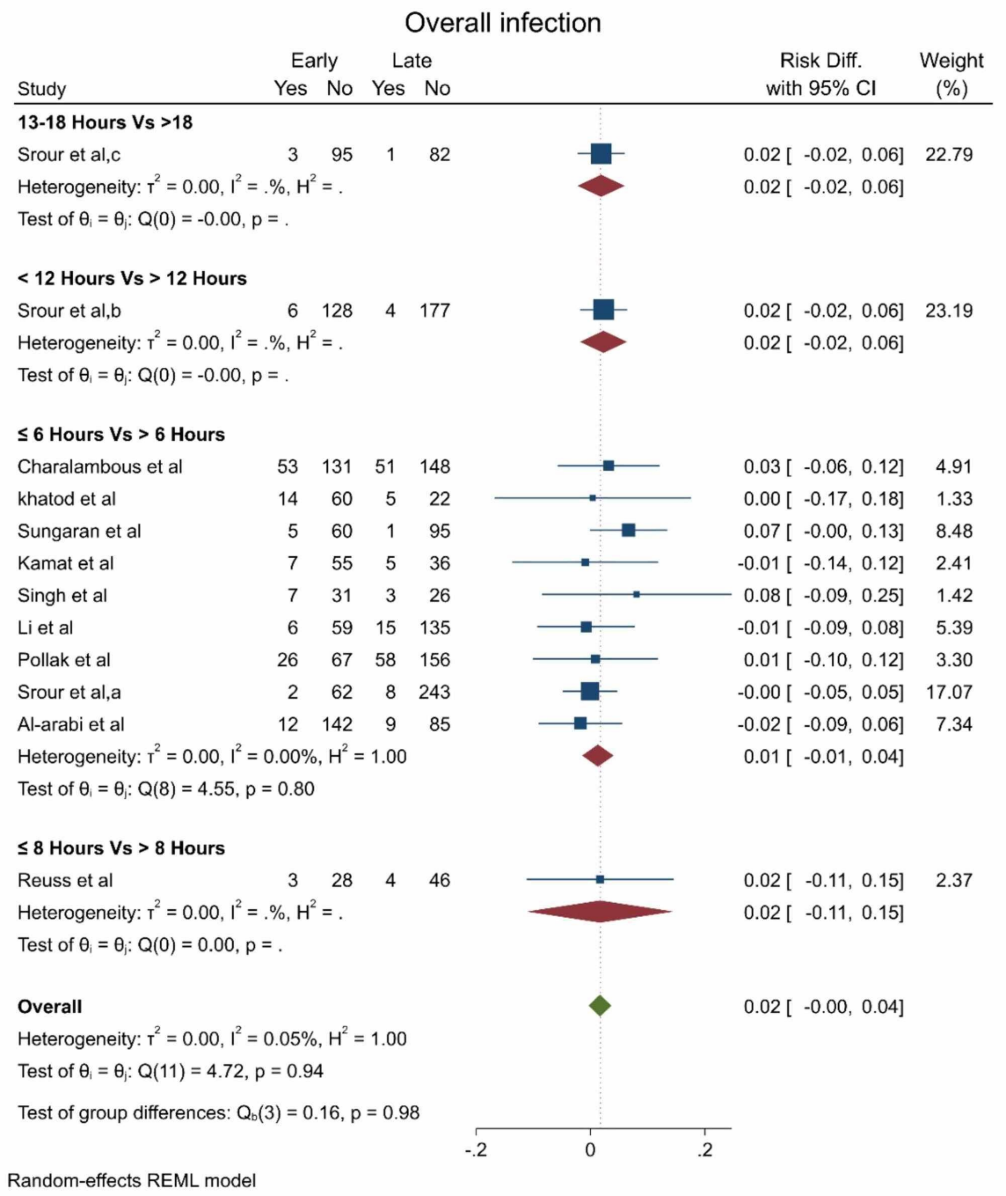
The overall infection rate

The pooled effect estimate showed no statistically significant difference between early and late debridement regarding the overall infection rate (RD 0.02, 95% CI [0 - 0.04], p = 0.94; Figure 2); there was no significant heterogeneity in the pooled estimate (I2 = 5%). The subgroup analysis showed that the non-significant difference was consistent regardless of the definition of early and late timing to debridement (Figure 2).

**Figure 3:**
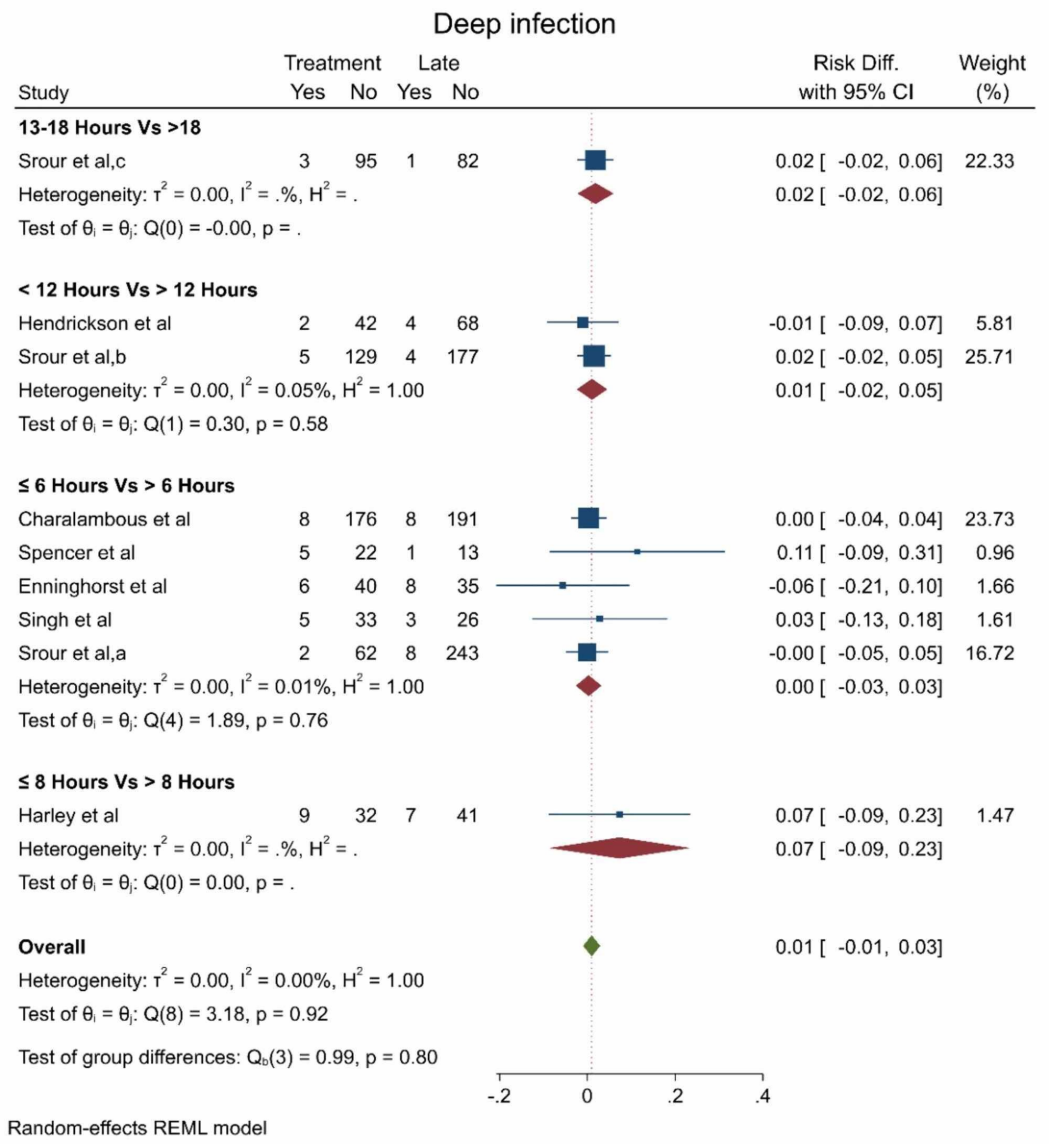
The deep infection rate

Likewise, the pooled effect estimate showed no statistically significant difference between early and late debridement regarding the deep infection rate (RD 0.01, 95% CI [-0.01 - 0.03], p = 0.92; Figure 3); there was no significant heterogeneity in the pooled estimate (I2 = 0%).

**Figure 4:**
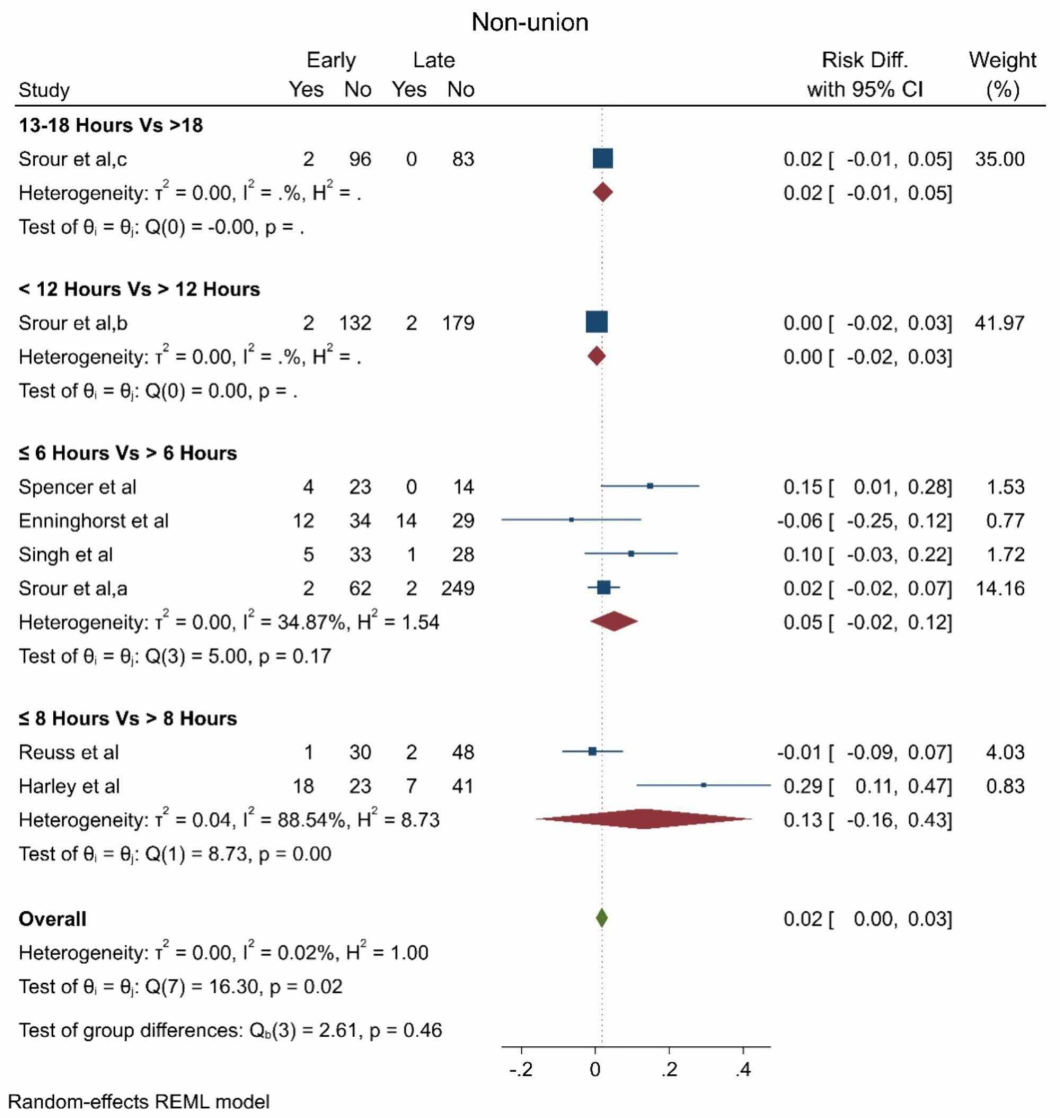
The nonunion rate

The pooled effect estimate showed no statistically significant difference between early and late debridement regarding the non-union rate as well (Figure 4).

**Figure 5:**
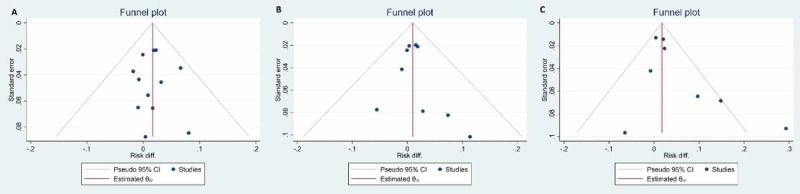
The funnel plots

The funnel plots showed little evidence of asymmetry by visual inspection (Figure 5).

## Discussion

Previously, it was commonly believed that open tibial fractures should undergo debridement and antibiotic administration with a maximum of six hours from injury; however, this concept has been challenged recently with a growing body of evidence. In this review, we aimed to provide updated evidence about the relation between the timing to debridement and the rate of infection. We found that the early debridement did not lead to a significant reduction in the risk of infection compared to late debridement. Besides, the pooled estimates showed no significant difference between early and late debridement regarding the deep infection and nonunion rates.

Infection is a major concern to orthopedic surgeons while treating open, long bone, fractures; it is a prevalent complication during the management of open fractures, especially with extensive tissue damage and contamination [20]. In the case of extensive or deep infection, the fracture can be complicated by the dysfunction of the limb, several organ failures, and mortality [3]. Thus, many strategies, mainly based on emergency measures, have been proposed to reduce the risk of infection in patients with long bone fractures. Early debridement within six hours from a fracture is the most widely accepted method for infection control in open fractures [21]. However, deriding the injury within six hours can be challenging in a real-life setting due to severe conditions [9]. Thus, previous reports have tried to investigate whether late debridement would significantly increase the risk of infection in patients with open fractures [24]. In this updated review, we demonstrated that late debridement did not lead to a significant increase in the risk of overall and deep infection rates among patients with open tibial fractures. This finding was consistent with a 2016 meta-analysis study, which showed a significant increase in the risk of infection in patients who underwent delayed surgical debridement (> 6 hours) [1]. In Schenker et al. systematic review, the risk of infection did not increase with delayed debridement [2]. The same findings were observed in the pediatric population [26]. The limited role of timing of debridement on the rate of infection can be explained by advances in antibiotics, fracture stabilization, and wound management strategies. Notably, our subgroup analysis demonstrated no significant increase in the risk of infection even when the debridement was delayed for 24 hours. In Srour et al. study, the patients, who underwent surgical debridement within 18-24 hours from injury, had similar infection rates to patients with earlier debridement [24]. The same results were reported by Li et al. [6].

While the present systematic review has the advantages of a comprehensive search of databases, homogeneity of pooled estimates, lack of substantial publication bias, and moderate-to-high quality of the included studies, we acknowledge the presence of some limitations. The findings of the present systematic review are mainly based on retrospective studies with their well-established limitations regarding misclassification and information biases. In addition, the data were limited to perform a meta-regression analysis in order to examine potential influencers of infection rate within the included studies. The inconsistencies in defining the infection and non-union, timing to surgery, and severity of fractures of included patients are other limitations.

## Conclusions

In conclusion, the current evidence demonstrates no impact of timing to surgical debridement on the infection rate following open tibial fractures in adult population. Our results demonstrated that the risks of infection, deep infection, and nonunion were similar between patients underwent delayed versus early debridement. Notably, these findings were consistent even when the delay extent to more than 12 hours after the injury. While emergent debridement within 24 hours is essential, the 6-hour rule should not be universally applied and the treating surgeons should consider several factors before deciding to urgently debride the wound within six hours from injury. Further, high-quality, evidence is still needed.

## Appendices

**Table 3:** Quality assessment of prospective studies

Author	Selection				Comparability		Outcomes			Score
	Outcome of interest not present at study start	Ascertainment of exposure	Representativeness of exposed cohort	Selection of the non-exposed cohort	Control for confounders	Comparability of groups on secondary risk factors	Adequacy of follow-up (loss)	Appropriate follow-up (length)	Assessment of outcomes	
Enninghorst et al.	*	*	*	*		*	*	*		7
Singh et al.	*	*	*	*	*	*	*	*		8
Fernandes et al.	*	*	*	*		*	*	*		7
Pollak et al.	*	*	*	*		*	*	*		7
Srour et al. (48.3%)	*	*	*	*	*	*	*	*		8
Al-Arabi et al. (< 50%)	*	*	*	*	*	*	*	*		8
	Yes *	Secure record (e.g. surgical records)*		Drawn from the same community as the exposed cohort *			Complete follow-up - all subjects accounted for	Yes*	Independent blind assessment *	
	No	Structured interview		Drawn from a different source				No	Record linkage *	
		Written self report		No description of the derivation of the non-exposed cohort					Self report	
		No description							No description	

**Table 4:** Quality assessment of retrospective studies

Author	Selection				Comparability	Exposure			Score
	Definition of controls	Selection of controls	Representativeness of the cases	Is the case definition adequate?	Comparability of cases and controls on the basis of the design or analysis	Non-response rate	Same method of ascertainment for cases and controls	Ascertainment of exposure	
Charalambous et al.	*		*	*	*	*	*	*	7
Khatod et al.	*	*	*	*	*	*	*	*	8
Spencer et al. (35%)	*		*	*	*	*	*	*	7
Sungaran et al.	*		*	*	*	*	*	*	7
Kamat et al.	*	*	*	*	*	*	*	*	8
Reuss and Cole	*	*	*	*	*	*	*	*	8
Harley et al.	*	*	*	*	*	*	*	*	8
Hendrickson et al.	*	*	*	*	*	*	*	*	8
Li et al.	*	*	*	*	*	*	*	*	8
	No history of endpoint (Infection) *	Community controls *	Consecutive or obviously representative series of cases *	Yes, with independent validation *		Same rate for both groups *	Yes *	Secure record (e.g. surgical records) *	
	No description of source	Hospital controls	Potential for selection biases or not stated	Yes, e.g. record linkage or based on self reports		Non-respondents described	No	Structured interview where blind to case/control status *	
		No description		No description		Rate different and no designation		Interview not blinded to case/control status	
								Written self report or medical record only	
								No description	
